# Variant analysis and PGT-M of *OTC* gene in a Chinese family with ornithine carbamoyltransferase deficiency

**DOI:** 10.1186/s12884-024-06696-5

**Published:** 2024-07-22

**Authors:** Yao Zhou, Xinxing Jiang, Yongfang Zhang, Yu Zhang, Fei Sun, Yanlin Ma

**Affiliations:** Hainan Provincial Key Laboratory for Human Reproductive Medicine and Genetic Research, Hainan Provincial Clinical Research Center for Thalassemia, Department of Reproductive Medicine, Key Laboratory of Reproductive Health Diseases Research and Translation, Hainan Medical University, Ministry of Education, the First Affiliated Hospital of Hainan Medical University, Hainan Medical University, Haikou, 570100 China

**Keywords:** *OTC*, Variant, PGT-M, Prenatal diagnosis

## Abstract

**Background:**

Ornithine carbamoyltransferase deficiency (OTCD) is a kind of X-linked metabolic disease caused by a deficiency in ornithine transcarbamylase leading to urea cycle disorders. The main reason is that the *OTC* gene variants lead to the loss or decrease of *OTC* enzyme function, which hinders the ammonia conversion to urea, resulting in hyperammonemia and severe neurological dysfunction. Here, we studied one Chinese family of three generations who consecutively gave birth to two babies with OTCD. This study aims to explore the pathogenicity of two missense variants in the *OTC* gene and investigate the application of preimplantation genetic testing for monogenic (PGT-M) for a family troubled by Ornithine carbamoyltransferase deficiency (OTCD).

**Methods:**

The retrospective method was used to classify the pathogenicity of two missense variants in the *OTC* gene in a family tortured by OTCD. Sanger sequencing was used to validate the variants in the *OTC* gene, and then the pathogenicity of variants was confirmed through family analysis and bioinformatics software. We used PGT-M to target the *OTC* gene and select a suitable embryo for transplantation. Prenatal diagnosis was recommended to confirm previous results using Sanger sequencing and karyotyping at an appropriate gestational stage. Tandem mass spectrometry (MS-MS) and gas chromatography-mass spectrometry (GC-MS) were used to detect fetal metabolism after birth. The number of the study cohort is ChiCTR2100053616.

**Results:**

Two missense variants, c.959G > C (p.Arg320Pro) and c.634G > A (p.Gly212Arg), were validated in the *OTC* gene in this family. According to the ACMG genetic variation classification criteria, the missense variant c.959G > C can be considered as “pathogenic”, and the missense variant c.634G > A can be regarded as “likely benign.” PGT-M identified a female embryo carrying the heterozygous variant c.959G > C (p.Arg320Pro), which was selected for transplantation. Prenatal diagnosis revealed the same variant in the fetus, and continued pregnancy was recommended. A female baby was born, and her blood amino acid testing and urine organic acid testing were regular. Follow-up was conducted after six months and indicated the girl was healthy.

**Conclusion:**

Our research first validated the segregation of both c.959G > C and c.634G > A variants in the *OTC* gene in a Chinese OTCD family. Then, we classified variant c.959G > C as “pathogenic” and variant c.634G > A as “likely benign”, providing corresponding theoretical support for genetic counseling and fertility guidance in this family. PGT-M and prenatal diagnosis were recommended to help the couple receive a female baby successfully with a six-month follow-up.

## Introduction

Ornithine carbamoyltransferase deficiency (OTCD, OMIM #311,250) is an X-linked inborn error of metabolism of the urea cycle, which occurs at a predictable frequency of 1 in 14,000 births [1]. This disease is the most common form of urea cycle disorders, accounting for 50% of the incidence rate of urea cycle disorders. Affected individuals show elevated levels of ammonia in their plasma, leading to severe neurological dysfunction and respiratory alkalosis [2–4]. OTCD is divided into neonatal onset and delayed onset (Age of onset > 28 days) in the clinic [5]. Neonatal onset often occurs in the male baby, with symptoms such as irritability, feeding difficulties, shortness of breath, and rapid progression to coma and respiratory failure, resulting in a high mortality rate [6]. Delayed onset is more common in infants and young children, with clinical manifestations such as growth and development disorders, hepatomegaly, behavioral abnormalities, and seizures. Chronic neurological damage is mainly present in children and adults and is characterized by irritability, mental disorders, intermittent vomiting, and behavioral abnormalities [7, 8].

The pathogenic gene for ornithine carbamyltransferase deficiency is the human *OTC* gene (OMIM* 300,461), founded on the short arm of the X chromosome with its cytogenetic location being Xp21.1 [9]. It has a total length of 73 kb with an open reading frame of 1,062 nucleotides and contains ten exons interjected by nine introns. *OTC* gene variant is an essential basis for the diagnosis of OTCD. More than 500 reported diseases associated variants in the *OTC* gene, of which 64.0% are missense or nonsense variants, 10.0% are splicing variants, 13.2% are small deletions or insertional variants, 9.8% are large deletions or repeats, 1.3% are complex variants with highly genetically heterogeneous [10], and no hotspot mutations have been reported. About 42% of mutations were associated with neonatal period onset, 21% with late-onset, and 37% with females [11–13]. Heterozygous women are mostly asymptomatic carriers or mild patients; hemi*zygous* male patients have more severe conditions.

Here, we analyzed a Chinese OTCD family and confirmed the pathogenicity of *OTC* gene variants through Sanger sequencing and genetic analysis. Additionally, PGT-M technology and prenatal diagnosis were used to guide the family to produce a healthy baby, which provides experience and a scientific basis for the prevention of birth defects in OTCD.

## Materials and methods

### Patients

A Chinese Han couple without hereditary diseases got married in 2014. The woman was pregnant in 2014 and gave birth to a boy at full term with a successful prenatal examination. The boy had difficulty feeding after birth and died prematurely on the 8th day without undergoing relevant testing. Then, the woman was pregnant again in 2015 and gave birth to a boy at full term. The boy experienced feeding difficulties, drowsiness, and poor reactions on the 5th day after birth. The boy underwent neonatal metabolic testing by tandem mass spectrometry (MS-MS) and gas chromatography-mass spectrometry (GC-MS) at The Sixth Affiliated Hospital of Sun Yat-sen University, and laboratory test results were as follows: Ala concentration 1373.79µmol/L↑ (normal, 50.00 to 400.00), Met concentration 62.37µmol/L↑ (normal, 8.00 to 50.00), Pip concentration 416.41µmol/L↑ (normal, 50.00 to 300.00), Pro concentration 2079.14µmol/L↑ (normal, 300.00 to 1800.00), Cit concentration 3.72µmol/L↓ (normal, 4.00 to 35.00) and Urine orotate analysis value 8.7225↑ (standard value, 0.033), indicating that the boy was an Ornithine carbamoyltransferase deficiency patient. Parents and the boy underwent *OTC* gene testing simultaneously, and the results showed that the proband carried a hemizygous variant c.959G > C (Arg320Pro) in the *OTC* gene. In comparison, the mother whom was a compound heterozygous for variants, c.959G > C (Arg320Pro) and c.634G > A (Gly212Arg), in the *OTC* gene. Unfortunately, the second baby died prematurely on the 9th day after birth. The couple continued to use contraception until 2019, when they went to the Reproductive Medicine Center at the First Affiliated Hospital of Hainan Medical University in Hainan, China. For genetic counseling, further guidance on childbirth was requested.

### Methods

#### Ethics statement

Informed consent was obtained from all participants. This study was approved by the institutional ethics committee of The First Affiliated Hospital of Hainan Medical University (Ethical approval number: 2023-KYL-264).

#### Study cohort

The number of the study cohort is ChiCTR2100053616 (11/25/ 2021). *OTC* gene was an X-linked recessive inheritance. Male members in the maternal family could provide substantial evidence. Therefore, the immediate and collateral relatives of the proband’s mother were included in the study. Except for the father of the proband, the father’s family members were not included in the study. Before the study, only the genotypes of the proband and parents were evident, while the genotypes of other members were unknown.

#### Sample collection and DNA extraction

Since the proband has died prematurely, and to obtain the co-separation of variation and family members, peripheral blood samples from both husband and wife, the wife’s parents, and the wife’s brothers and sisters were collected with EDTA-K2 anticoagulation. Using the Qiagen DNA Extraction Kit (Qiagen), extract genomic DNA according to the instructions and store it at -20 ℃ for future use.

#### Variant validation

Sanger sequencing was conducted to validate and detect the variant on the *OTC* gene and identify variants on both spouses and family members. We used Primer 3.0 software to design the *OTC* gene (NM_000531.6): amplification of upstream and downstream sequences at c.634 and c.959 loci. Primers were synthesized by Shanghai Shenggong Technology Co., Ltd. The primer sequences are *OTC*-F1: tacgcctggatttcatctcc, *OTC*-R1: gggctggtaacgtaacctaaa. *OTC*-F2: tgccacatataatagtcaaaaagtgg, *OTC*-R2: catgagcaagtaatgtagttgga. The PCR amplification products were sequenced using an ABI3500 sequencer (Applied Biosystems, USA) and Seqman software Chromas 2.6.6 (Technelysium, South Brisbane, QLD, Australia) was used to view Sanger sequencing results and compare them with human genome sequences.

#### Pathogenicity analysis of variants

The sequencing data were aligned to the *OTC* reference genome on NCBI NM_000531.6. Various disease databases were consulted to assess the pathogenicity of the variants: HGMD Pro, Clinvar, SNP/ExAC, gnomAD, and 1000 Genomes Project databases. SIFT, Mutation Taster, Revel, PolyPhen-2, and PROVEAN bioinformatics software were used to predict protein function for mutation sites. At the same time, pathogenic analysis of variants was conducted according to the genetic variation interpretation standards and guidelines released by the American Society for Medical Genetics and Genomics (ACMG) in 2015. Alpha Fold 2 was used to model the structure of *OTC*, predicting the changes in protein spatial structure on missense mutations, and visualizing the three-dimensional structure of the protein using PyMOL 2.3 software.

#### PGT-M and prenatal diagnosis

After conducting the pathogenicity of the variants, the couples expressed their knowledge and requested to have healthy offspring through PGT-M. Controlled ovarian hyperstimulation was conducted with the use of a gonadotropin-releasing hormone (GnRH) agonist based on a long protocol to obtain enough oocyte. Oocyte retrieval was performed 35–36 h after hCG injection, and all metaphase II oocytes underwent intracytoplasmic sperm injection (ICSI). All embryos were cultured to the blastocyst stage. When embryos reached the blastocyst stage, assisted hatching was performed to create a circular opening in the zona pellucida for trophectoderm (TE) biopsy. 5–10 TE cells were aspirated with a biopsy pipette, and the specimens were cleaved. Embryo vitrification was performed using the Kitazato vitrification cryotop method. TE cells were washed in PBS (with 0.1% HAS) and transferred to the PCR tube. The whole genome of the cell was amplified by the Pico PLEX single-cell WGA kit (NEB-WGA) according to the manufacturer’s protocol. The sequencing library was constructed by a personalized genome library-building Kit (zykw-c-003) produced by Peking Jabrehoo Med Tech Co. Ltd. Sequencing was performed with the Illumina Miseq System. The method of embryo detection uses NGS to construct haplotypes of SNPs targeting the *OTC* gene.

The PGT results were divided into three situations: embryos that didn’t carry the variant could be transplanted, male hemizygous were not allowed, and female heterozygous were considered carriers. Due to the random inactivation of the X chromosome, transplantation can only be carried out after sufficient genetic consultation with the patient. After the patient transferred the embryo and got pregnant, a prenatal diagnosis was recommended to confirm previous results. At the gestational age of 17 weeks and beyond, 30mL of the fetal amniotic fluid was collected from the mother. About 15 ml amniotic fluid for karyotyping and fetal DNA extraction from the remaining amniotic fluid was performed using a QIAamp DNA Mini Kit (Qiagen, Germany), following the manufacturer’s protocols. Sanger sequencing was used to investigate both missense variants c.959G > C and c.634G > A in *OTC* gene by forward and reverse sequencing. The sequencing results were analyzed according to previously established procedures.

#### Neonatal examination and follow-up

Detailed prenatal and postpartum ultrasound examinations were conducted to eliminate the chance of fetal malformations after a successful pregnancy. Subsequently, neonatal metabolic testing was done after birth, and a six-month follow-up of our offspring was conducted.

## Results

### Variant validation

**Fig. 1:**
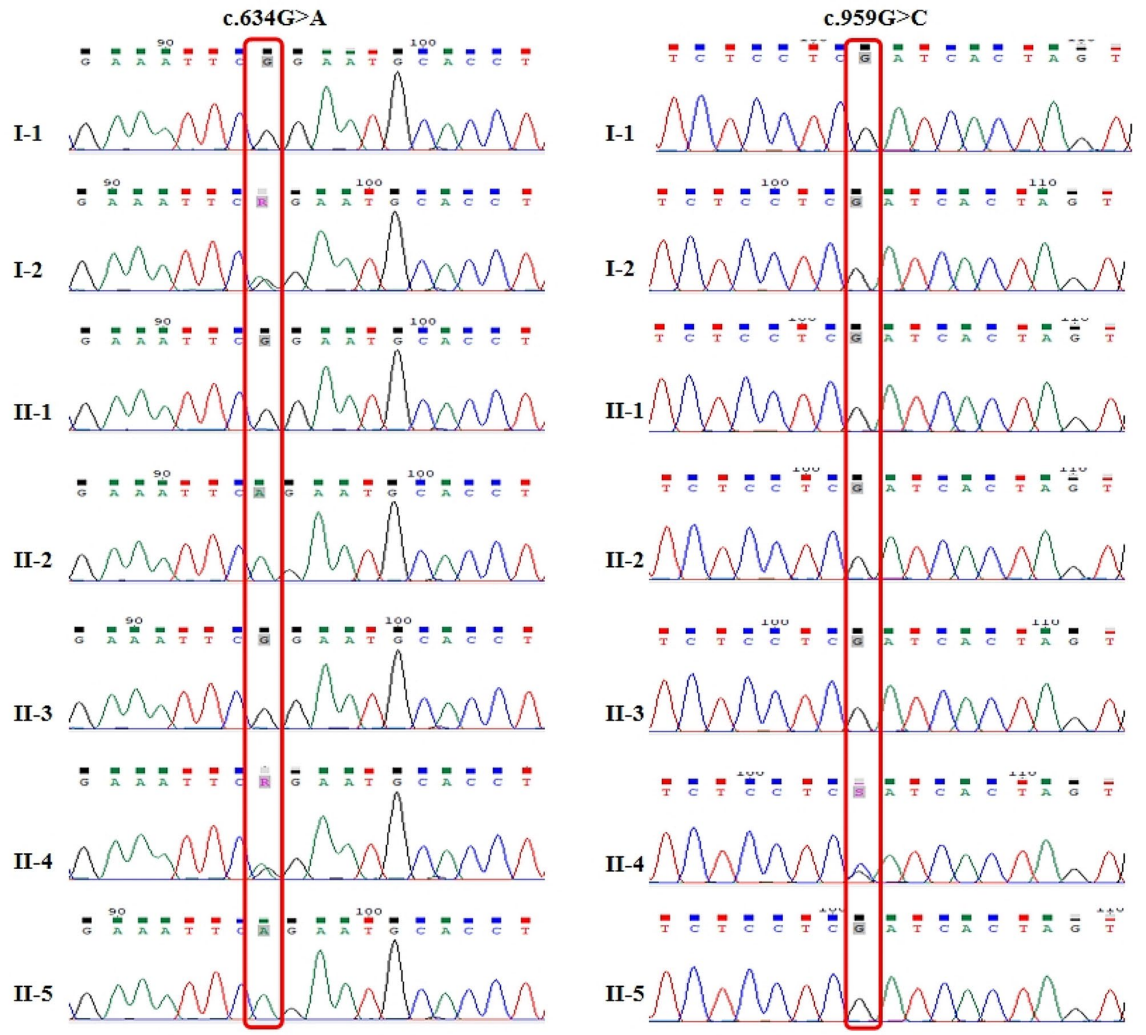
Sanger sequencing revealed that the variation occurred in the *OTC* gene at the c.634 and c. c.959 sites. The mother of proband is compound heterozygous, while the c.959G > C is a de novo mutation

**Fig. 2:**
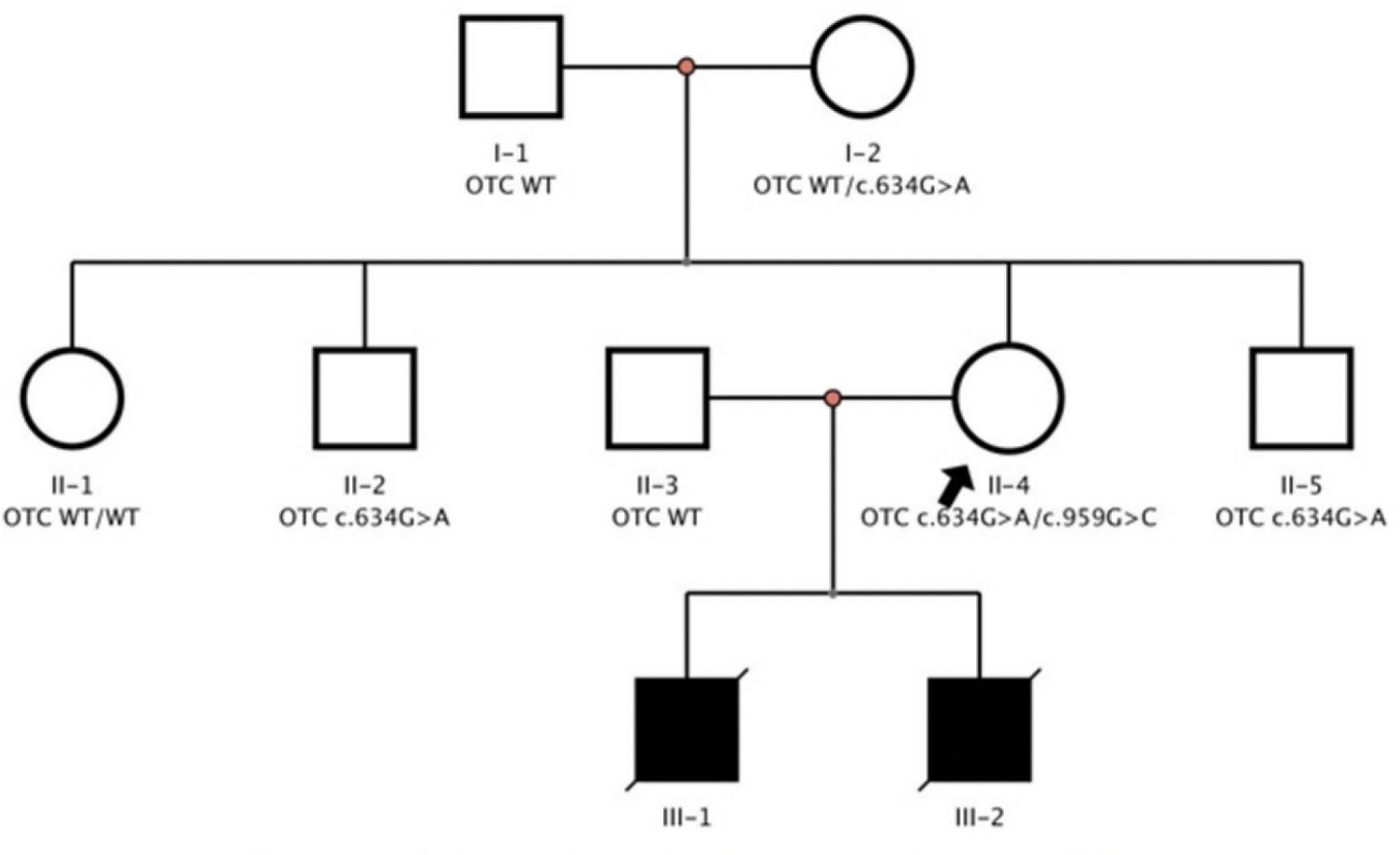
Pedigree analysis of this Chinese family. I-1: Grandfather of the proband; I-2: Grandmother of the proband, carried a heterozygous variant of c.634G > A; II-1: Aunt of the proband; II-2, II-5: Two uncles of the proband, carried the same hemizygous variant of c.634G > A; II-3: Father of the proband; II-4: Mother of the proband, carried two compound heterozygous variants of c.959G > C and c.634G > A; III-1: Brother of the proband, without test; III-2: The proband in this family, tested by another unit instead of this study. The black arrow represents the member carried a de novo variant of c.959G > C in the family (II-4). A filled circle represents a male OTCD patient, an unfilled square (male), and a circle (female) represents healthy individuals

In this Chinese family, two missense variants c.959G > C (p. Arg320Pro) and c.634G > A (p. Gly212Arg) were validated in *OTC* gene by Sanger sequencing and the familial segregation of variants in the family members were confirmed (Table 1). The grandfather (I-1) and aunt (II-1) of the proband (III-2) did not carry c.959G > C (p. Arg320Pro) and c.634G > A (p. Gly212Arg) variants. The grandmother (I-2) had a heterozygous mutation of c.634G > A (p. Gly212Arg), and the uncles (II-2, II5) were hemizygous with c.634G > A (p. Gly212Arg), but no OTCD phenotype was observed. The father (II-3) was wild type, and the mother (II-4) was compound heterozygous due to two variants in the same gene. Unexpectedly, the female carrying c.959G > C (p. Arg320Pro) was a de novo variant and occurred in trans, which was confirmed by the female’s parents as biological parents but did not carry this variant (Figs. 1 and 2; Table 1).

**Table 1:** Variants in OTC gene in the proband’s family

Members	Age	Sex	c.634G > A *p*.Gly212Arg	c.959G > C *p*.Arg320Pro	OTCD
Grandfather (I-1)	58	M	WT	WT	N
Grandmother (I-2)	61	F	Het	WT	N
Aunt (II-1)	37	F	WT	WT	N
Uncle (II-2)	36	M	Hemi^mat^	WT	N
Father (II-3)	34	M	WT	WT	N
Mother (II-4)	33	F	Het^mat^	Het^dn^	N
Uncle (II-5)	29	M	Hemi^mat^	WT	N
Brother (III-1)	8 day	M	NT	NT	P
Proband (III-2)	9 day	M	WT*	Hemi*	P

Abbreviations: dn, de novo; mat, maternal; WT, wild type; F, female, M, male; NT, not tested; *, tested by another unit instead of this study; P, patient; N, normal

### Pathogenicity analysis of variants

**Fig. 3:**
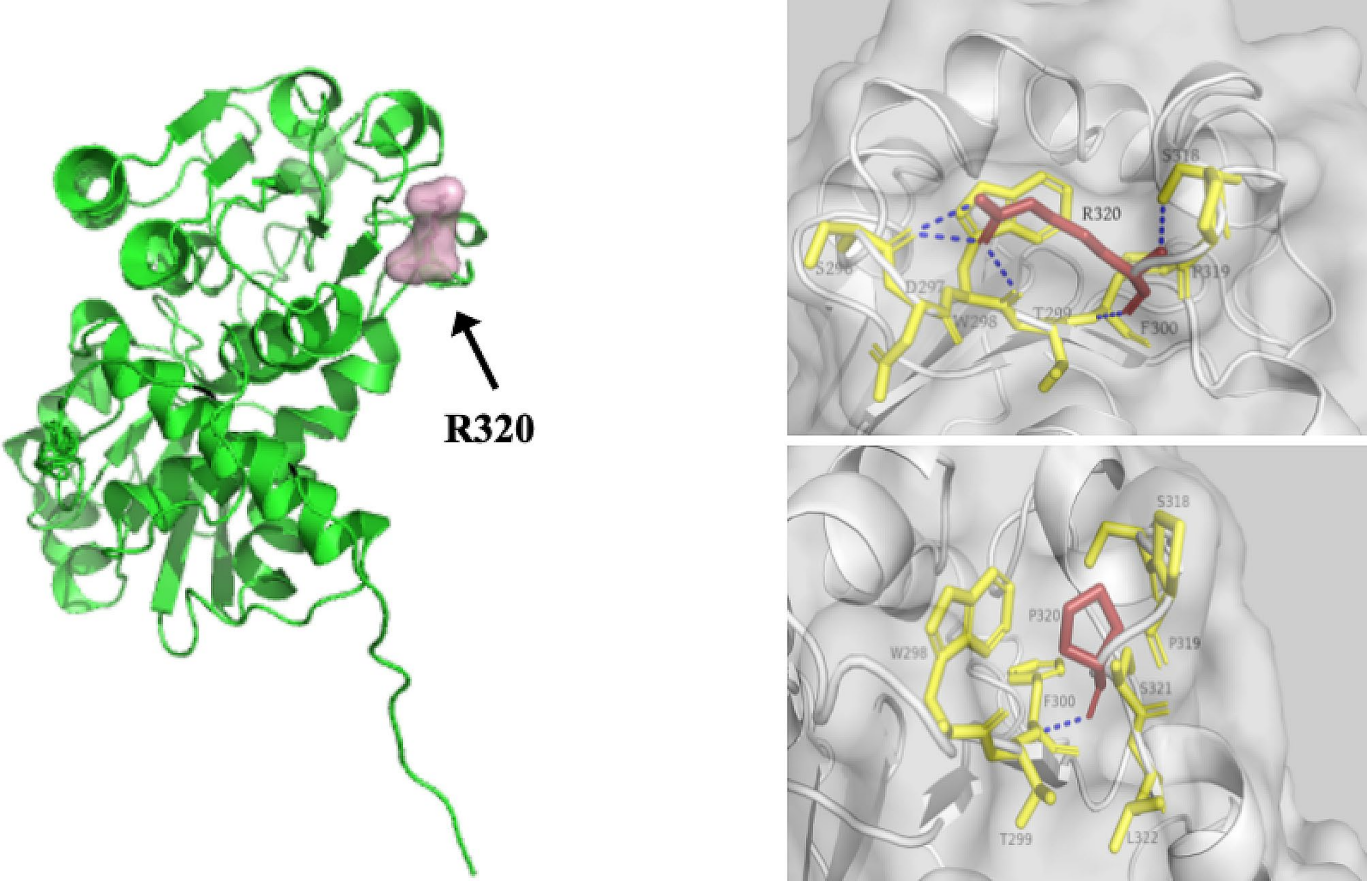
The structure of *OTC* was modeled to predict the effect of missense mutations on protein structure using Alpha Fold 2. The arrow on the left shows the location of amino acid site 320, and the hydrogen bonds between the amino acids and changes in protein spatial structure are shown on the right. Blue dotted lines are hydrogen bond interactions

The missense variant c.959G > C on exon 9 of the *OTC* gene can cause the 320th amino acid arginine to be replaced by proline. Silico analysis such as PolyPhen 2, SIFT, Vest3, REVEL, and PROVEAN predict that the encoded proteins are harmful or degraded (Table 2). Protein model analysis showed that when arginine at position 320 is replaced with proline, the hydrogen bonds and hydrophobic interactions between the substituted amino acids change, resulting in changes in the protein’s spatial structure (Fig. 3). The frequency of this variant is absent in the average control population of the ExAC and gnomAD databases. Pedigree analysis in this family confirmed the missense variant c.959G > C as de novo occurred on the mother (II-4) of the proband. Based on the above results, according to the ACMG genetic variation classification criteria, the missense variant c.959G > C can be considered pathogenic.

**Table 2:** In silico analysis for predicting the pathogenicity of variant c.959G > C

Predicting Tools	Score	Predicting Results
SIFT	0.002	Damaging
Polyphen2	0.989	Probably_damaging
PROVEAN	-4.84	Damaging
VEST3	0.872	Damaging
REVEL	0.658	Damaging

The missense variant c.634G > A on exon 6 of the *OTC* gene can cause the 212th amino acid glycine to be replaced by arginine. The frequency of this mutation is 0.0000457 in the average control population of the ExAC database and 0.00002803 in the average control population of the gnomAD database. This mutation is recorded as likely benign in the ClinVar database. The two males (II2, II5) in the family carrying the hemizygous variant are healthy adults. According to the ACMG genetic variation classification criteria, the mutation is judged as potentially benign (BS2 + BP5).

### PGT-M results and clinical outcome

**Fig. 4:**
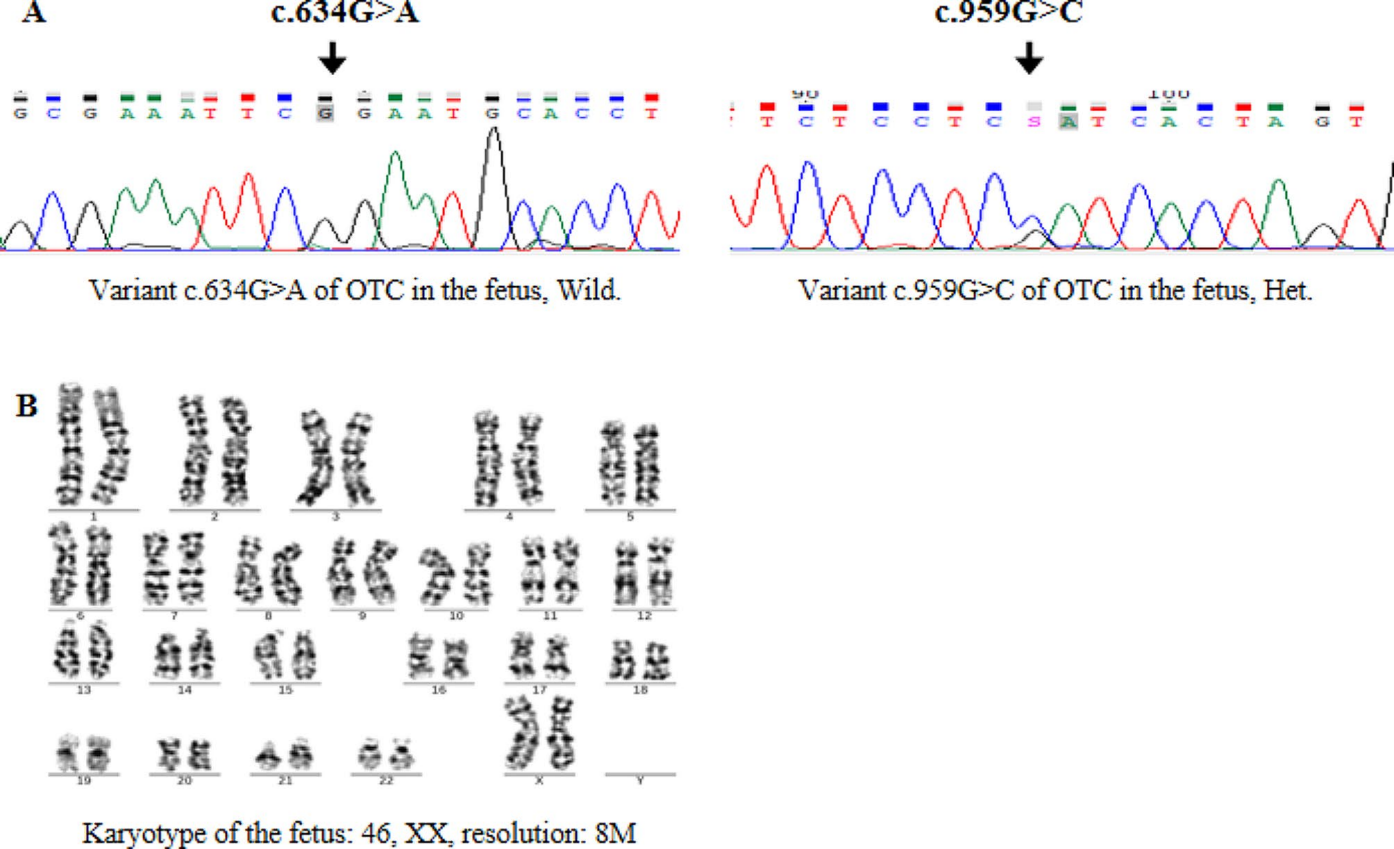
The results of prenatal diagnosis. **(A)** Sanger sequencing revealed that the fetus carrying the heterozygous c.959G > C variant in the *OTC* gene. **(B)** The results of the G-banding karyotype analysis in the fetus showed a resolution of 46, XX, and 8 M

**Fig. 5:**
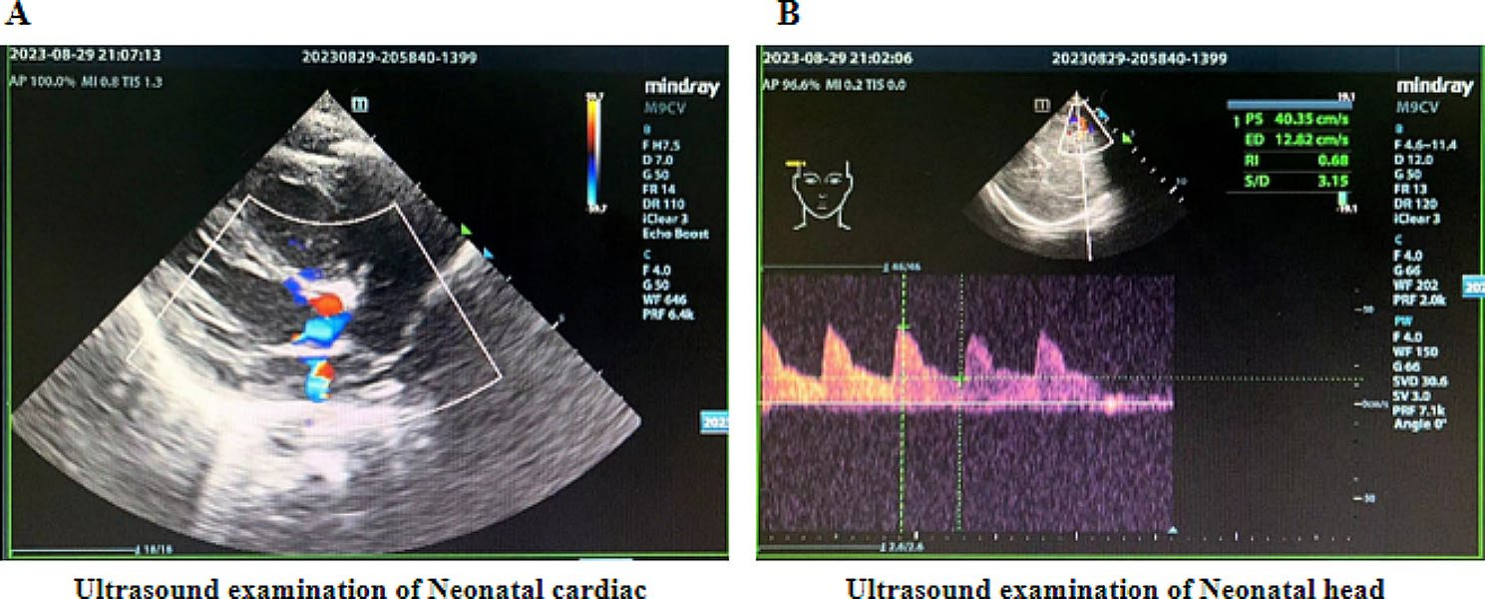
Neonatal ultrasound examination. **(A)** The ultrasound examination of the newborn’s heart showed no abnormalities. **(B)** The ultrasound examination of the newborn’s head shows no abnormalities

Finally, due to poor ovarian response in the patient, only one oocyte at the MII stage was obtained through controlled ovarian hyperstimulation. Fortunately, after testing, the remaining embryo was identified as a female embryo carrying heterozygous variant, which can be used for transplantation. Although females will be carriers, parents should be informed about this issue and have genetic counseling regarding symptomatic female cases. After a thorough consultation with the patient, in December 2022, the patient transplanted the embryo carrying the heterozygous variant of c.959G > C in the *OTC* gene and finally obtained a clinical pregnancy. At 18 weeks of gestation, the patient underwent prenatal diagnosis; sequencing of the fetal amniotic cell’s DNA revealed that the fetus carrying the heterozygous variant of c.959G > C in the *OTC* gene, and the karyotype was 46, XX (Fig. 4). The prenatal diagnosis results were consistent with the PGT results. Thus, continued pregnancy was recommended. During the full pregnancy period, all ultrasonographic examinations did not indicate any morphological fetal abnormality. The patient gave birth to a girl at full term at the First Affiliated Hospital of Hainan Medical University in August 2023. No abnormalities were found in the newborn’s ultrasound postnatal examination (Fig. 5) and neonatal metabolic testing by MS-MS and GC-MS were as follows: Ala concentration 172.7µmol/L (normal, 100.00 to 450.00), Met concentration 21.95µmol/L (normal, 9.50 to 45.00), Pro concentration 930.88µmol/L (normal, 450.00 to 2700.00), Cit concentration 5.90µmol/L (normal, 5.00 to 30.00) and Urine orotate analysis value was 0 (standard value, 0.033). So far, follow-ups at the age of half a year have indicated successful results.

## Discussion

Ornithine carbamoyltransferase deficiency, also known as hyperammonemia type II, is a kind of metabolic disease caused by urea cycle disorders [14]. OTCD patients can develop symptoms at any age and are generally divided into neonatal onset and delayed onset based on the onset time. The onset time is related to the degree of enzyme activity deficiency. Newborn onset patients usually have a complete loss of *OTC* enzyme activity, mostly male hemizygous mutations [6], mainly manifested as vomiting, refusal to eat, drowsiness, convulsions, hypotonia, and delayed psychomotor development. Heterozygous women are mostly asymptomatic carriers or mild patients.

*OTC* gene (NM_000531.5) encodes a 354-amino acid protein, which is expressed mainly in the liver and secondly in the intestinal mucosal cells [2]. *OTC* enzyme can catalyze the formation of ornithine and carbamyl phosphate in mitochondria, citrulline, the latter is transported to the cytoplasm to continue participating in the ornithine cycle [15]. The ornithine cycle is the main pathway of ammonia degradation in the organism. When the *OTC* gene undergoes pathogenic mutations, it can lead to a decrease or loss of *OTC* enzyme activity, which in turn leads to inhibition of citrulline synthesis and ornithine cycle, ammonia degradation disorders, and an increase in blood ammonia levels [2, 10]. Excessive accumulation of ammonia has strong central nervous system toxicity, leading to acute or chronic encephalopathy, neurological and psychiatric damage [10].

*OTC* gene mutations are an important basis for the diagnosis of OTCD [16]. There are currently over 500 reported pathogenic mutations in *OTC* gene, of which 64% are missense or nonsense mutations, 10% are splicing mutations, 13.2% are small segment deletions or insertion mutations, and 9.8% are large deletion or duplication mutations, 1.3% are complex mutations [10], without hot spot mutations. In the present study, a Chinese family that had given birth to two children with OTCD was investigated. Two missense variants c.959G > C and c.634G > A were validated in *OTC* gene by Sanger sequencing. Through pedigree analysis, two healthy adult males in this family were confirmed to be c.634G > A hemizygotes. It was reported for the first time that two adult males in the same family were hemizygous with variation c.634G > A, providing strong evidence of benign grading, indicating that the variant c.634G > A was likely benign, consistent with the recorded submitted in the Clinvar database. Wel et al. collected clinical and genotypic data and possible enzyme activity and X chromosome inactivation profiles from a cohort of 289 women carrying *OTC* mutations and 197 related males [17]. For the first time, a Gly212Val variant at the exact location was reported. Conservative prediction suggests that Gly at this location is highly conserved and indicates that the mutation is pathogenic. This may be due to Val having stronger hydrophobicity, while Gly and Arg exhibit hydrophilicity, which may have different effects on the tendency of secondary structure, leading to varying degrees of harm caused by changes in other amino acids at the same position. Furthermore, it is interesting to note that when we used prediction software to predict the Gly212Val and Gly212Arg variants separately, most software predictions showed that both Gly212Val and Gly212Arg were harmful. However, in our study, the co-segregation of families showed that the adult male hemizygous carrying the Gly212Arg variant did not exhibit any OTCD-related phenotypes, and three hemizygous individuals were also observed in the gnomAD database. This may suggest that the evidence of co-segregation in the family is more favorable than predicted by bioinformatics software under some conditions. More importantly, the variant c.959G > C carried by the proband inherited from his mother, but the mother’s parents did not have the variant, which was confirmed to be de novo. This situation met the PS2 evidence in the ACMG guidelines, which is considered strong evidence. In addition, the pathogenicity of variant c.959G > C was indirectly confirmed through conservation analysis and protein function prediction. According to the ACMG guidelines, this mutation has been classified as pathogenic, consistent with the record submitted in the Clinvar database. However, there was no more detailed description of this variant in the Clinvar database before.

In this case, the variant c.959G > C in the *OTC* gene was the cause of the proband’s illness, suggesting couples have another child through PGT-M. PGT is an ideal option to reduce the risk of birth of an affected baby without facing termination of pregnancy. PGT for monogenic disorders has been successfully applied to high-risk couples for more than three decades [18]. A study suggests that 0.8 -1% of European couples are at risk of having children with autosomal recessive inherited diseases [19], and these couples can prevent their offspring from developing diseases through prenatal diagnosis or PGT. However, carrier screening has also brought many problems to PGT-M, such as the degree of pathogenic variation that leads to PGT-M intervention. Too many screening targets may lead to no embryos available for transplantation. In addition, multi-gene disease risk assessment is also a new challenge faced by PGT-M. In 2019, Treff [20] extended PGT-M to predict the risk of polygenic disease for the first time and established an SNP prediction model for type 1 diabetes to provide a reference for embryo transfer, which caused great controversy. There is a lack of specific regulatory guidelines for PGT of multi-gene diseases. A series of issues, such as how to construct a multi-gene disease risk prediction model for different ethnic groups and how to evaluate its clinical efficacy, still need to be explored.

Similarly, as the current clinical detection of embryos relies on invasive biopsy techniques to obtain embryonic cell materials, the question of whether this invasive procedure will affect embryonic development and even cause birth defects has always been a concern. These issues have posed challenges to the consultation and implementation of PGT-M. A study suggests the overall prevalence of birth defects in IVF-ICSI pregnancies was 118.6/10,000 births, and the most common congenital anomaly was cardiac malformation [21]. Other studies have also shown that, compared to hormone stimulation, the manipulation of gametes by IVF/ICSI may be the main factor contributing to abnormal cardiovascular metabolism in offspring [22]. Apart from mechanical procedures that involve damage to the embryo, other factors, such as exposure to nonphysiologically environments and ice crystals produced by embryo freezing, can potentially increase the risk to the fetus. So, when facing whether patients need to obtain pregnancy through PGT-M, there should be sufficient indications and detailed communication, as well as effective preventive measures during the pregnancy process.

This study confirmed that the female carried a de novo heterozygous variant c.959G > C, classified the variant c.959G > C as “pathogenic”. The variant c.959G > C in the *OTC* gene was the cause of the proband’s illness, and there was a high risk for this couple, with a 1/4 chance of conceiving an OTCD male baby again. PGT-M technology can directly assist this couple in avoiding the risk of re-pregnancy with *OTC* children or iatrogenic-induced abortion. Unfortunately, due to the patient’s low ovarian response, only one oocyte at the MII stage was obtained and cultured into a blastocyst. The test result showed that the embryo was a female carrier. OTCD is often fatal in affected males with progressive hyperammonemia, cerebral edema, liver and renal failure, and early death if untreated [23]. Heterozygous females show variable clinical manifestations, both in terms of the onset of the disease and its severity. They are influenced by the genotype and the proportion of hepatocytes with the wild-type X chromosome inactivated [24]. In our study, the patient only had one female carrier embryo that could be transferred. After sufficient genetic counseling, the patient was willing to transfer this embryo and agreed to undergo invasive prenatal diagnosis and routine ultrasound monitoring during pregnancy. As expected, the fetus showed no abnormalities during pregnancy, and the prenatal diagnosis supported the result of PGT-M. After the fetus’s birth, the ultrasound evaluation of the newborn had no cardiac malformation (Fig. 5), and the metabolic testing of blood ammonia and urine orotate was normal, indicating that the baby should be a carrier of *OTC* gene mutations rather than a patient. During the follow-up at six months, the baby had good growth and development, which once again confirmed our results.

The limitation of the research was that the study of variations has yet to be able to achieve functional validation at the cellular level or in model animals. In addition, there was a lack of relevant tests for the girl’s random X chromosome inactivation. However, the results of metabolic and ultrasound tests indicate that the girl was not an OTCD patient. In this study, we have described variant c.634G > A as “likely benign” through co-segregation of genotype and phenotype in the family. However, as reported in the literature above, the pathogenic causes of different amino acids at the same position were very significant. Regarding mutation c.959G > C, we modeled the structure of *OTC* and predicted the changes in protein spatial structure between the mutation and wild type. The three-dimensional structure analysis of the protein confirmed the pathogenicity of the variant c.959G > C, and the evidence remains “pathogenic”. This may require us to further refine it through functional studies at the cellular level or using model animals.

## Conclusions

In conclusion, we reported a Chinese OTCD family with two missense variants in the *OTC* gene by Sanger sequencing and genetic analysis, first validating the segregation of both c.959G > C and c.634G > A variants in an *OTC* family. Then we classified variant c.959G > C as “pathogenic” and variant c.634G > A as “likely benign”, providing corresponding theoretical support for genetic counseling and fertility guidance in this family. We predict the effect of missense mutations on the protein structure of the *OTC* using Alpha Fold 2. PGT-M and prenatal were recommended to help the couple receive a female baby successfully.

## Data Availability

Sequence data that support the findings of this study have been deposited in the ClinVar with the submission ID SUB14172826 and SUB14172848.

## Abbreviations

OTCD: Ornithine carbamoyltransferase deficiency
ACMG: American Society for Medical Genetics and Genomics
GnRH: Gonadotropin-releasing hormone
TE: Trophectoderm
PGT-M: Preimplantation genetic testing-monogenic disease
WES: Trio-whole exome sequencing
SNP: Single nucleotide polymorphism
MS-MS: Tandem mass spectrometry
GC-MS: Gas chromatography-mass spectrometry
